# X-linked chronic granulomatous disease combined with disseminated nontuberculous mycobacteria and fungal infections: Diagnosis and treatment analysis

**DOI:** 10.1097/MD.0000000000046453

**Published:** 2025-12-26

**Authors:** Wenyuan Huang, Yuxuan He, Lu Zhan, Xianming Shi

**Affiliations:** a Department of Pediatrics, Hangzhou Red Cross Hospital, Hangzhou, Zhejiang Province, China; b Department of Pediatrics, The Second Clinical Medical College, Zhejiang Chinese Medical University, Hangzhou, Zhejiang Province, China; c Department of Pediatrics, Lanxi Hospital of Traditional Chinese Medicine, Lanxi, Zhejiang Province, China.

**Keywords:** CYBB gene mutation, disseminated nontuberculous mycobacterial disease, fungal infection, hematopoietic stem cell transplantation, X-linked chronic granulomatous disease

## Abstract

**Rationale:**

X-linked chronic granulomatous disease (X-CGD) is a rare genetic immunodeficiency, predominantly affecting males. It leads to defective neutrophil function and increased susceptibility to infections, particularly nontuberculous mycobacteria and fungi. Early diagnosis is challenging due to its nonspecific symptoms.

**Patient concerns:**

A 1-year-5-month-old male presented with recurrent lymphadenopathy, erythematous and ulcerative skin lesions, and persistent fever. These symptoms, along with pulmonary involvement, raised concerns for infectious diseases, leading to a misdiagnosis of tuberculosis.

**Diagnoses:**

Genetic testing revealed a hemizygous mutation in the CYBB gene, confirming the diagnosis of X-CGD. Further investigations identified *Mycobacterium gordonae* and *Candida parapsilosis* as the causative pathogens.

**Interventions:**

The patient received broad-spectrum antibiotics and antifungal therapy, including meropenem, clarithromycin, linezolid, and voriconazole. Immunomodulatory treatments, including intravenous immunoglobulin and interferon-γ, were initiated to support immune function. Hematopoietic stem cell transplantation was planned for long-term management.

**Outcomes:**

The patient showed significant improvement, with regression of cervical and pulmonary lesions. Ongoing recovery was observed, with plans for stem cell transplantation to achieve a potential cure.

**Lessons:**

X-CGD should be suspected in cases with recurrent infections and unusual responses to treatment. Genetic testing is essential for confirmation, and early initiation of prophylactic and targeted therapies can improve outcomes. Awareness of atypical presentations is crucial for timely diagnosis and intervention.

## 1. Introduction

Chronic granulomatous disease (CGD) is a rare primary hereditary immunodeficiency disorder, with an incidence of approximately 1 in 2,00,000.^[1,2]^ Over 60% of CGD cases are X-linked (X-CGD), which primarily results from mutations in the CYBB gene encoding the nicotinamide adenine dinucleotide phosphate (NADPH) oxidase subunit.^[3]^ Notably, X-CGD has a unique susceptibility to both nontuberculous mycobacterial (NTM) and fungal infections, which are less commonly observed in other immunodeficiencies. Several studies have emphasized the particular vulnerability of X-CGD patients to these infections, with clinical reports detailing the challenges in diagnosis and treatment.^[4–6]^ X-CGD typically manifests in early childhood and predominantly affects males. The clinical presentation is characterized by recurrent, severe bacterial and fungal infections along with granuloma formation in various tissues, most commonly affecting the lymph nodes, subcutaneous tissues, lungs, liver, and gastrointestinal tract.^[7]^ The nonspecific nature of these clinical features often leads to delayed or missed diagnoses.

We treated a rare case of X-CGD in which the patient presented with recurrent lymphadenitis and pulmonary infection. Initially, this patient was misdiagnosed with tuberculosis. Later, it was confirmed that he had a disseminated NTM infection complicated by a fungal infection.

## 2. Case report

The patient, a 1 year and 5 months old, was admitted to the hospital on August 19, 2022 due to “swelling of the left neck for 4 days.” The outpatient department initially diagnosed “cervical lymphadenitis,” but there was no improvement after 4 days of oral cefaclor. At 4 months of age, after BCG vaccination, the patient developed an abscess in the left arm along with swelling and abscess formation in the left axillary lymph nodes; an axillary lymph node aspirate culture yielded no mycobacterial growth, and this was diagnosed as a “BCG adverse reaction.” Family history revealed that the mother had a history of tuberculous pleurisy and that an older brother had died in early childhood.

**Figure 1.**
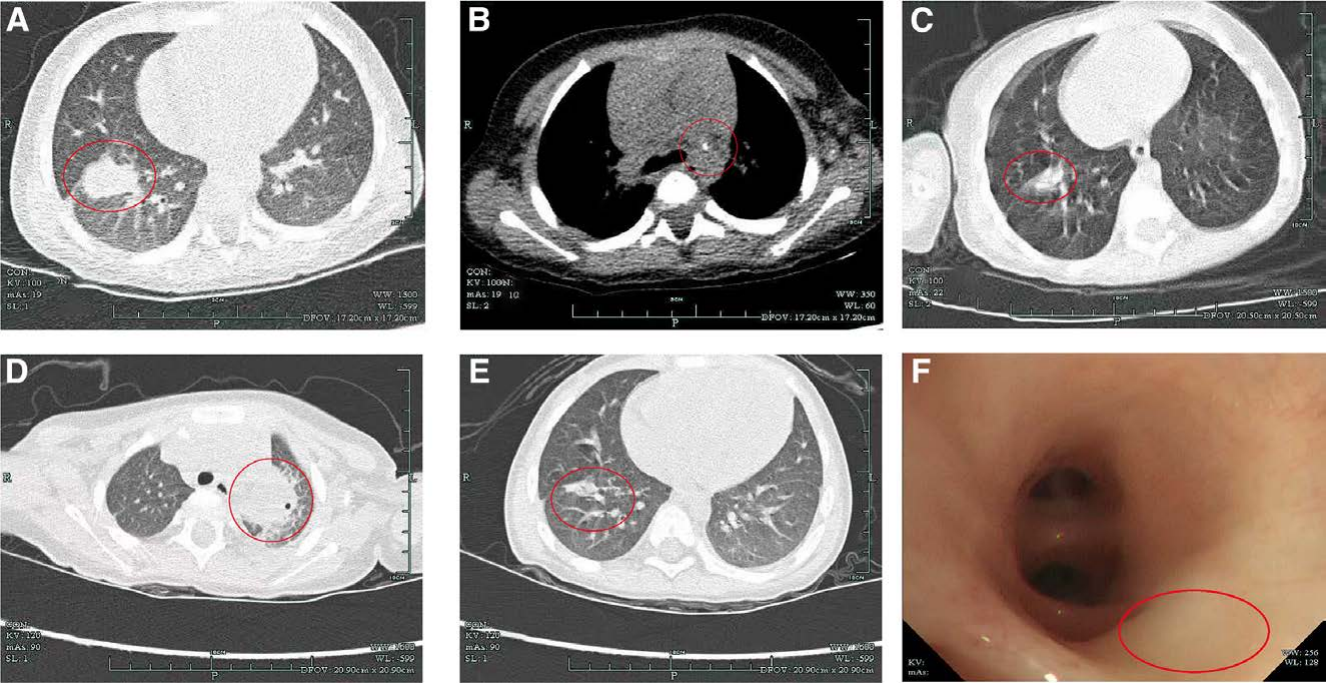
Serial chest computed tomography (CT) and bronchoscopy findings of the patient. (A, B) Chest CT image obtained on August 23, 2022, showing initial pulmonary lesions. (C) Follow-up CT scan on October 17, 2022, indicating disease regression. (D, E) CT scan on July 31, 2023, demonstrating the emergence of new lesions. (F) Bronchoscopy image taken on October 27, 2022, showed mild mucosal swelling in the anterior basal segment of the right lower lobe, revealing airway abnormalities consistent with the CT findings. CT = computed tomography.

Upon admission, physical examination revealed multiple enlarged lymph nodes on the left side of the neck, characterized by limited mobility, medium consistency, tenderness, and overlying erythema and swelling. A 1-cm incision was noted in the left axilla, without active exudation. Cardiopulmonary and abdominal examinations were unremarkable. Laboratory tests showed elevated white blood cell count, C-reactive protein, and accelerated erythrocyte sedimentation rate. The tuberculin skin test was strongly positive, whereas the T-cell spot test for tuberculosis infection, the acid-fast bacilli (AFB) smear, and *Mycobacterium tuberculosis* DNA in sputum were all negative. Chest computed tomography (CT) on August 23, 2022 revealed infectious lesions in the right lower lobe and calcification of mediastinal lymph nodes (Fig. 1A, B). An initial diagnosis of primary pulmonary tuberculosis with cervical lymph node tuberculosis was made, and antituberculosis therapy with isoniazid (H), rifampicin (R), and pyrazinamide (Z) was initiated.

Two months later, the patient was readmitted due to worsening pain and enlargement of the cervical mass. Examination revealed significant enlargement of the left cervical mass, poorly defined margins, nodal confluence, limited mobility, considerable tenderness, and overlying skin inflammation. Follow-up chest CT on October 18, 2022 showed partial absorption of the right lower lung lesion (Fig. 1C) although the cervical lymphadenopathy had progressed. Given the lack of response to standard anti-TB therapy, disseminated BCG disease was suspected, and linezolid was added to the regimen. During this period, the patient experienced recurrent fevers, left cervical lymphadenitis, and ulceration. Bronchoscopy revealed mild mucosal swelling in the anterior basal segment of the right lower lobe (Fig. 1F). BALF tested negative for *M tuberculosis* DNA and NTM DNA.

**Figure 2.**
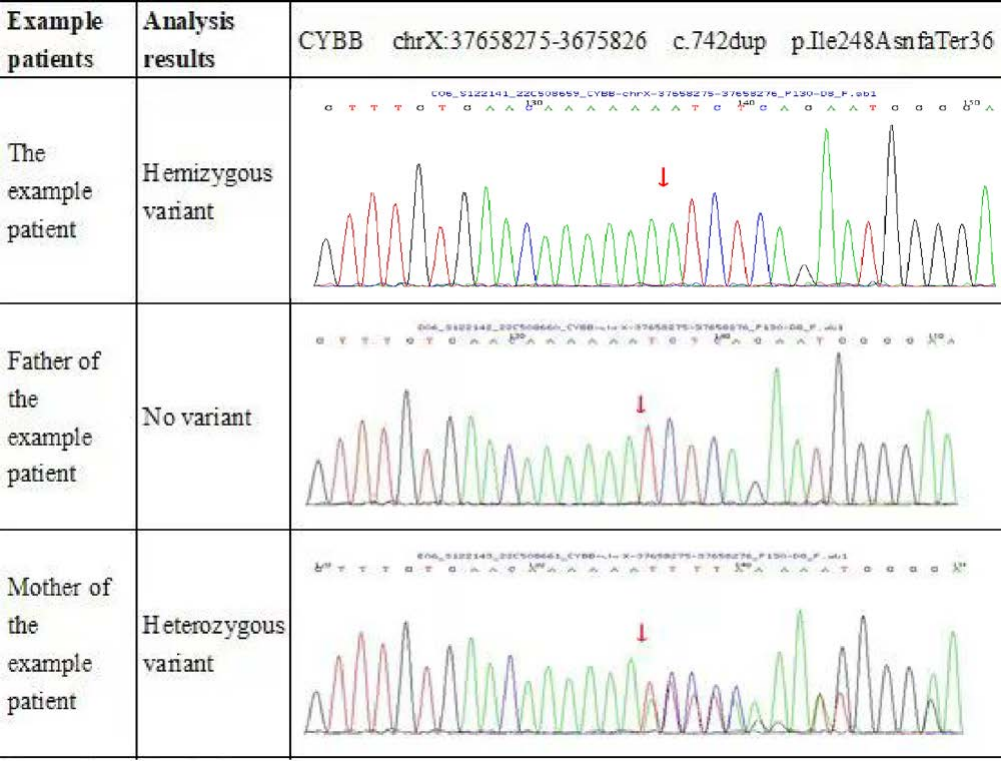
Genetic testing results of the patient and his biological parents. The figure displays the sequencing data of the proband, his father, and his mother. Detected variants are highlighted and annotated, showing the hereditary pattern and confirming the presence of the mutation in the patient.

Despite ongoing anti-TB therapy after discharge, the patient continued to experience cervical lymphadenopathy and systemic inflammatory symptoms (fever, elevated white blood cell count, and elevated C-reactive protein). Considering the atypical course of the disease, genetic testing via whole-exome sequencing was performed and revealed a hemizygous mutation in the CYBB gene in the patient and a heterozygous variant at c.742dup (p.Ile248AsnfsTer36) in his mother (Fig. 2). These findings confirmed the diagnosis of X-CGD. Subsequent cultures of BALF identified *Mycobacterium gordonae*, which was resistant to first-line anti-TB agents, and prompted a switch to clarithromycin and linezolid for targeted NTM treatment.

On July 9, 2023, this patient was readmitted with fever and worsening cervical lymphadenopathy. Examination revealed fused, suppurative lymph nodes with skin ulceration on the left side of the neck. Chest CT demonstrated a mass in the left upper lobe, raising suspicion of an infectious lesion or lung abscess (Fig. 1D, E). Working diagnoses included disseminated NTM infection, X-CGD, and possible lung abscess. Treatment included meropenem for broad-spectrum antibacterial coverage, clarithromycin and linezolid for NTM, fluconazole for fungal prophylaxis, and intravenous immunoglobulin (IVIG) for immune support. Due to persistent fever and clinical deterioration, the patient was transferred to a children’s hospital for further management.

A biopsy of the left cervical lymph node tissue showed chronic suppurative granulomatous inflammation. Next-generation sequencing of the biopsy tissue detected 1,83,019 sequences (reads) of Candida parapsilosis, and no other pathogens were identified. A neutrophil respiratory burst assay showed a markedly reduced activation rate of 0.34% (normal > 90%; Table 1), consistent with a diagnosis of X-CGD. Voriconazole was initiated for antifungal therapy. Simultaneously, cotrimoxazole (SMZ) was prescribed for bacterial prophylaxis, and interferon-γ (IFN-γ) along with intermittent IVIG were given as supportive therapy.

**Table 1:** Neutrophil respiratory burst test.

Test indicators	Results	Units	Reference range	Test method
Control the neutrophil activation rate	0.08	%	<10%	Flow cytometry
Neutrophil activation rate after stimulation	0.34	%	>90%	Flow cytometry
Stimulation index (SI)	0.78	–	>100	Calculation method

SI = stimulation index.

Final diagnoses included X-CGD, disseminated NTM infection, and deep fungal infection. However, central nervous system infection was excluded. Currently, the cervical and pulmonary lesions have regressed, and this patient is recovering well. Umbilical cord blood stem cell transplantation is being planned. Detailed information is provided in Table S1, Supplemental Digital Content, https://links.lww.com/MD/Q854.

## 3. Discussions

### 3.1. Pathogenesis

The pathogenesis of X-CGD primarily stems from dysfunction of the NADPH oxidase complex and results from mutations in the CYBB gene. The CYBB gene locates on chromosome Xp21.1, encodes the gp91phox protein, and is a crucial component of the NADPH oxidase complex.^[8]^ This enzyme is essential for generating superoxide radicals within phagocytes and is vital for microbial killing. Mutations in CYBB, including missense, nonsense, splice-site, and deletion mutations, lead to the absence or malfunction of gp91phox and disrupt the assembly and activity of the NADPH oxidase complex.^[9]^ In this case, this patient carried a missense mutation in CYBB. The dysfunctional NADPH oxidase impairs the production of reactive oxygen species and compromises the bactericidal and fungicidal functions of phagocytes. Consequently, patients with X-CGD are particularly susceptible to catalase-positive pathogens such as *Staphylococcus aureus*, Salmonella, and Aspergillus.^[10]^ In some countries like India and China, pulmonary infections with Aspergillus and *M tuberculosis* are frequently reported among X-CGD patients.^[11,12]^ In this case, this patient developed disseminated NTM and fungal infections. This presentation is atypical and may contribute to diagnostic delays. Moreover, NADPH oxidase dysfunction disrupts inflammatory regulation and cellular signaling, which exacerbates immune dysregulation.^[13]^

### 3.2. Clinical manifestations

X-CGD presents with a broad-spectrum of clinical features, and the most notable features are recurrent, severe bacterial and fungal infections. X-CGD typically manifests during infancy or early childhood, and its common infection sites include the lungs, skin, lymph nodes, liver, and bones. Among these, the lungs are the most frequently affected site. Pulmonary manifestations range from pneumonia to lung abscesses and empyema.^[14]^ Cutaneous presentations include cellulitis, abscesses, and suppurative lymphadenitis. In this case, this patient experienced recurrent infections of the skin, lymph nodes, and lungs. Liver abscesses, often caused by *S. aureus* or gram-negative bacilli, are also common.^[15]^ A hallmark of X-CGD is granuloma formation which affects multiple organ systems. Gastrointestinal granulomas can lead to obstruction, diarrhea, or malabsorption,^[16]^ while genitourinary involvement may cause urinary tract obstruction or renal dysfunction. Additional findings in children with X-CGD may include growth retardation, anemia, thrombocytosis, and other nonspecific signs.^[17]^ Some patients also present with autoimmune phenomena such as systemic lupus erythematosus or inflammatory bowel disease, and these phenomena are likely related to immune dysregulation from impaired NADPH oxidase function.^[18]^ Disease severity often correlates with residual NADPH oxidase activity, and patients with partial activity may exhibit milder phenotypes.^[19]^

### 3.3. Treatment

Management of X-CGD focuses on infection prophylaxis and immunomodulation. Prophylactic administration of SMZ (cotrimoxazole) and itraconazole significantly reduces infection rates.^[20]^ IFN-γ therapy enhances residual NADPH oxidase activity and boosts host immune defense.^[21]^ For active infections, early and targeted use of broad-spectrum antibiotics and antifungal agents is critical. In this case, delayed initiation of antifungal therapy and IFN-γ following definitive diagnosis may have contributed to disease progression.^[18]^ Hematopoietic stem cell transplantation (HSCT) remains the only curative option and is recommended for patients with severe disease and an available HLA-matched donor.^[22]^ Emerging evidence suggests that gene therapy may offer a promising alternative in the future.^[23]^ To facilitate early identification and management of complications, long-term follow-up should include periodic assessments of growth, nutritional status, and organ function.^[24]^

### 3.4. Experience and lessons learned from diagnosis and treatment

The diagnostic and therapeutic journey of this case provided important clinical insights into managing X-CGD. Initially, this patient’s adverse reaction to BCG vaccination should have immediately prompted an evaluation for immunodeficiency, including immune function assays and neutrophil respiratory burst test. The triad of an adverse BCG reaction, recurrent infections, and a family history of early death in infancy due to an undetermined cause should have been recognized as a “red flag” for a potential immunodeficiency. During the patient’s initial hospitalization, a history of their older brother’s death in infancy due to an undetermined cause was documented. However, this case was not thoroughly investigated, which may have led to an oversight of a potential heritable immunodeficiency. Although the patient had a strongly positive tuberculin skin test test and chest CT findings (a right lower lobe lesion with mediastinal lymph node calcification) suggestive of infection, his T-cell spot test for tuberculosis infection and sputum *M tuberculosis* DNA tests were negative; nonetheless, a diagnosis of primary pulmonary tuberculosis was made, primarily based on the mother’s history of tuberculous pleurisy. The discordant T-SPOT result was not adequately considered. Functional testing, such as the neutrophil respiratory burst test, should have been initiated immediately to expedite the diagnosis, rather than waiting for genetic sequencing. Because of an inadequate response to antituberculosis therapy and the detection of low immunoglobulin levels, an immunodeficiency was suspected, leading to whole-exome sequencing. Despite considering disseminated BCG disease and arranging bronchoscopy, the more accessible neutrophil respiratory burst test was not utilized initially. This case reinforces the critical importance of initiating prophylaxis against NTM and fungi immediately upon clinical suspicion of CGD, particularly given the plan for HSCT. After identifying *M gordonae* in BALF and confirming a hemizygous CYBB mutation, anti-NTM therapy was adjusted. However, the opportunistic characteristic of *M. gordonae* should have warranted concurrent prophylaxis with SMZ and itraconazole, as well as the initiation of IFN-γ therapy to enhance immune function. Such interventions might have potentially averted the progression to invasive fungal disease. In the setting of a severe fungal infection, broader infection surveillance should be undertaken, with particular attention to excluding central nervous system involvement.

## 4. Conclusion

X-CGD is a rare X-linked inherited immunodeficiency, which predominantly affects males and may initially present as abnormal reactions to BCG vaccination. Typical clinical features include recurrent respiratory tract infections, lymphadenitis, and cutaneous abscesses. Genetic testing is crucial for a definitive diagnosis. After diagnosis, it is necessary to vigilantly monitor infectious foci, promptly provide targeted anti-infective therapy, and proactively prevent bacterial and fungal infections to reduce morbidity. Adjunctive therapies, such as IVIG replacement and IFN-γ, are recommended to enhance immune defense. For eligible patients, HSCT, pursued after achieving control overactive infections, offers a potential cure and the prospect of improved long-term outcomes.

## Acknowledgments

The authors take full responsibility for the content, analysis, and conclusions of the case report, and no clinical or medical decisions were influenced by the AI tool.

## Author contributions

**Conceptualization:** Wenyuan Huang, Xianming Shi.

**Data curation:** Wenyuan Huang, Yuxuan He, Lu Zhan.

**Formal analysis:** Wenyuan Huang.

**Methodology:** Wenyuan Huang.

**Project administration:** Wenyuan Huang.

**Supervision:** Xianming Shi.

**Writing – original draft:** Wenyuan Huang.

**Writing – review & editing:** Wenyuan Huang, Xianming Shi.

## Supplementary Material



## References

[1] Jianxin HE Shunying Z Zaifang J. Clinical features and CYBB mutation analysis in children with X-linked recessive chronic granulomatous disease. J Chin Clin Pediatr. 2011;29:41–5.

[2] Rider NL Jameson MB Creech CB. Chronic granulomatous disease: epidemiology, pathophysiology, and genetic basis of disease. J. Pediatric Infect. Dis. Soc.. 2018;7(suppl_1):S2–5. 29746675 10.1093/jpids/piy008 PMC5946813

[3] Bousfiha A Jeddane L Picard C. Human inborn errors of immunity: 2019 update of the IUIS phenotypical classification. J Clin Immunol. 2020;40:66–81. 32048120 10.1007/s10875-020-00758-x PMC7082388

[4] Bassiri-Jahromi S Doostkam A. Fungal infection and increased mortality in patients with chronic granulomatous disease. J Mycol Med. 2012;22:52–7. 23177814 10.1016/j.mycmed.2011.12.079

[5] Sadaf H Zhao B Lelenwa LC. Granulomatous fungal and non-tuberculous mycobacterial infestation complicating chronic lung disease: outcomes in patients undergoing lung transplantation. Ann Diagn Pathol. 2021;55:151832. 34628284 10.1016/j.anndiagpath.2021.151832

[6] Falcone EL Holland SM. Invasive fungal infection in chronic granulomatous disease: insights into pathogenesis and management. Curr Opin Infect Dis. 2012;25:658–69. 22964947 10.1097/QCO.0b013e328358b0a4

[7] Kalotychou V Mermigkis D Kanariou MG. *Pneumocystis jirovecii* pneumonia in a X-linked chronic granulomatous disease female carrier. Idcases. 2021;26:e01323. 34786342 10.1016/j.idcr.2021.e01323 PMC8577472

[8] Thomsen IP Smith MA Holland SM Creech CB. A comprehensive approach to the management of children and adults with chronic granulomatous disease. J Allergy Clin Immunol Pract. 2016;4:1082–8. 27178966 10.1016/j.jaip.2016.03.021

[9] Yu D Wang W Zheng Y Shen K. Study progress of chronic granulomatous disease in children. Chin J Appl Clin Pediatr. 2020;35:877–80.

[10] Roos D. Chronic granulomatous disease. Br Med Bull. 2016;118:50–63. 26983962 10.1093/bmb/ldw009 PMC5127417

[11] Rawat A Vignesh P Sudhakar M. Clinical, immunological, and molecular profile of chronic granulomatous disease: a multi-centric study of 236 patients from India. Front Immunol. 2021;12:625320. 33717137 10.3389/fimmu.2021.625320 PMC7946827

[12] Gao LW Yin QQ Tong YJ. Clinical and genetic characteristics of Chinese pediatric patients with chronic granulomatous disease. Pediatr Allergy Immunol. 2019;30:378–86. 30716179 10.1111/pai.13033 PMC6850071

[13] Yu HH Yang YH Chiang BL. Chronic granulomatous disease: a comprehensive review. Clin Rev Allergy Immunol. 2021;61:101–13. 32524254 10.1007/s12016-020-08800-x

[14] Chiu TL Leung D Chan KW. Phenomic analysis of chronic granulomatous disease reveals more severe integumentary infections in X-linked compared with autosomal recessive chronic granulomatous disease. Front Immunol. 2021;12:803763. 35140711 10.3389/fimmu.2021.803763 PMC8818666

[15] Goldblatt D Thrasher AJ. Chronic granulomatous disease. Clin Exp Immunol. 2000;122:1–9. 11012609 10.1046/j.1365-2249.2000.01314.x PMC1905749

[16] López-Hernández I Suárez-Gutiérrez M Santos-Chávez EE Espinosa S Blancas-Galicia L. Chronic granulomatous disease. Update and review. Rev Alerg Mex. 2019;66:232–45. Enfermedad granulomatosa crónica. Actualización y revisión. 31200421 10.29262/ram.v66i2.577

[17] Kohn DB Booth C Kang EM. Lentiviral gene therapy for X-linked chronic granulomatous disease. Nat Med. 2020;26:200–6. 31988463 10.1038/s41591-019-0735-5 PMC7115833

[18] Roos D van Leeuwen K Hsu AP. Hematologically important mutations: X-linked chronic granulomatous disease (fourth update). Blood Cells Mol Dis. 2021;90:102587. 34175765 10.1016/j.bcmd.2021.102587

[19] Köker MY Camcioğlu Y van Leeuwen K. Clinical, functional, and genetic characterization of chronic granulomatous disease in 89 Turkish patients. J Allergy Clin Immunol. 2013;132:1156–63.e5. 23910690 10.1016/j.jaci.2013.05.039

[20] Slack MA Thomsen IP. Prevention of infectious complications in patients with chronic granulomatous disease. J. Pediatric Infect. Dis. Soc.. 2018;7(suppl_1):S25–30. 29746681 10.1093/jpids/piy016 PMC5946879

[21] Hodny Z Reinis M Hubackova S Vasicova P Bartek J. Interferon gamma/NADPH oxidase defense system in immunity and cancer. Oncoimmunology. 2016;5:e1080416. 27057461 10.1080/2162402X.2015.1080416 PMC4801460

[22] Gul Y Hazar E Kapakli H. Chronic granulomatous disease: a single-center experience in Central Anatolia. Pediatr Neonatol. 2025;66:134–41. 38918167 10.1016/j.pedneo.2024.02.008

[23] Kaufmann KB Chiriaco M Siler U. Gene therapy for chronic granulomatous disease: current status and future perspectives. Curr Gene Ther. 2014;14:447–60. 25245086 10.2174/1566523214666140918113201

[24] Liese J Kloos S Jendrossek V. Long-term follow-up and outcome of 39 patients with chronic granulomatous disease. J Pediatr. 2000;137:687–93. 11060536 10.1067/mpd.2000.109112

