# Supplementary material for: X-linked chronic granulomatous disease combined with disseminated nontuberculous mycobacteria and fungal infections: Diagnosis and treatment analysis
Source: Medicine (Baltimore). 2025 Dec 26;104(52):e46453. doi: 10.1097/MD.0000000000046453 (PMC12746978; doi:10.1097/MD.0000000000046453)
Supplement: Supplementary file 1 [file medi-104-e46453-s001.docx]

**Table S1**. Diagnosis and Treatment Process

| **Time** | **Clinical event** | **Examination finding** | **Diagnosis/treatment adjustment** | **Critical turning point** |
| --- | --- | --- | --- | --- |
| Apr. 2022 | Abscess in the left arm after BCG vaccination | Axillary lymph node puncture fluid tuberculosis culture: negative | Diagnosed as "BCG adverse reaction" | The first immune abnormal signal |
| Aug. 2022 | Admission with a complaint of "swelling in the left neck for 4 days" | - Enlarged cervical lymph nodes with elevated skin temperature  -WBC/CRP/ESR↑  -PPD (+++)  -T-Spot (-)  - Lung CT: Infection foci in the lower lobe of the right lung, mediastinal lymph node calcification | Preliminary diagnosis: Lymph node tuberculosis + pulmonary tuberculosis  . Start the 2HRZ/4HR regimen | Misdiagnosed as tuberculosis |
| Oct. 2022 | Swelling and pain in the neck worsen | - Re-examination of lung CT: Partial absorption of the lesion in the right lung  - Persistent enlargement of cervical lymph nodes  - Persistent WBC/CRP ↑ | Poor anti-tuberculosis effect → Add linezolid | Suspected disseminated BCG disease |
| Nov. 2022 | Recurrent fever and skin ulcers in the neck | - Bronchoscopy: Swelling of the mucosa in the right lower lobe  - TB/NTM DNA in BALF (-)  - Low immunoglobulin | Continue anti-tuberculosis treatment (poor response) | Suspected immunodeficiency disease |
| Jan. 2023 | Whole exome sequencing | CYBB gene c.742dup mutation (hemizygous)  Mother is a heterozygous carrier | Diagnosis of X-CGD | The genetic diagnosis is clear. |
| Feb.  2023 | Bronchoalveolar lavage culture for non-tuberculous mycobacteria. | Isolation of Gordonia species from bronchoalveolar lavage culture (resistant to first-line anti-tuberculosis drugs). | Diagnosis of non-tuberculous mycobacterial infection; cessation of anti-tuberculosis treatment and initiation of clarithromycin + linezolid therapy. | Identification and drug susceptibility testing of NTM species. |
| Jul. 2023 | Fever again, suppurated and ruptured lymph nodes in the neck | - Chest CT: Mass (abscess?) in the upper lobe of the left lung  - BALF culture: Mycobacterium Gordon (resistant to first-line anti-tuberculosis drugs) | Stop the anti-tuberculosis drug and switch to clarithromycin + linezolid anti-(NTM) fluconazole prophylaxis + immunoglobulin support | Confirm NTM infection and suspect fungal infection |
| Aug. 2023 | Referred to Zhejiang Provincial Children's Hospital | - Lymph node pathology: Chronic suppurative granulomatosis  -NGS: Candida sequence | - Voriconazole (Antifungal)  - Anti-NTM treatment  - SMZ prevents bacterial infection +IFN-γ immunomodulation | Confirm Coexisting fungal infections |
| Feb. 2025 | Prepare for umbilical cord blood transplantation | - Absorption of neck and lung lesions  - Regularly assess infection control and immune status | Plan HSCT | Radical treatment preparation |
